# Role of Magnesium, Effects of Hypomagnesemia, and Benefits of Magnesium Supplements in Cardiovascular and Chronic Kidney Diseases

**DOI:** 10.7759/cureus.64404

**Published:** 2024-07-12

**Authors:** Raju Panta, Subash Regmi

**Affiliations:** 1 Physiology and Pathology, Burrell College of Osteopathic Medicine, Melbourne, USA; 2 Critical Care Medicine, University of Southern Carolina, Columbia, USA

**Keywords:** benefits of magnesium supplements, magnesium supplements, hypomagnesemia in chronic kidney diseases, hypomagnesemia in cardiovascular diseases, chronic kidney diseases, cardiovascular diseases, role of magnesium, magnesium

## Abstract

Cardiovascular diseases (CVDs) account for nearly half of chronic kidney disease (CKD)-related deaths. Hypomagnesemia has been associated with various cardiovascular conditions and predicts a decline in renal function leading to end-stage renal disease (ESRD). The objective of this review is to delve into and discuss the significance of magnesium (Mg) in cardiovascular and renal functions, the clinical consequences of hypomagnesemia on CVD and CKD, and the benefits of Mg supplementation in managing CVD and CKD. This review is the result of an extensive search for pertinent articles in databases like PubMed, Medline, PubMed Central, and Google Scholar. Based on the literature search conducted in this review, we concluded that Mg protects against various CVDs and delays the progression of CKD. Mg can regulate pathways associated with inflammation, oxidative stress, and fibrosis. Therefore, maintaining slightly elevated Mg levels and timely Mg supplementation may benefit patients with CVD and CKD. There is a need for additional prospective randomized controlled trials to fully comprehend the therapeutic effects of Mg on CVD and CKD along with setting individualized target levels for serum Mg in such patients.

## Introduction and background

Cardiovascular diseases (CVDs) are responsible for almost half of chronic kidney disease (CKD)-related mortalities [1]. Conversely, CKD significantly increases the risk of developing cardiovascular issues, highlighting the interrelated nature of these conditions. Approximately 13.4% of the world's population suffers from CKD [2], with hypertension and diabetes being the leading causes [3,4]. Effective management of these conditions can improve both renal and cardiovascular health outcomes, potentially delaying or preventing the progression to end-stage renal disease (ESRD) [5]. Hypomagnesemia has been associated with various cardiovascular conditions, including hypertension, cardiomyopathy, cardiac arrhythmia, atherosclerosis, dyslipidemia, and diabetes [6]. Hypomagnesemia predicts the loss of renal function in established CKD [7]. Hypomagnesemia is linked with a decline in renal function and progression to ESRD [8,9]. This review aims to explore and discuss the role of magnesium (Mg) in cardiovascular and renal functions, the clinical impact of hypomagnesemia on CVD and CKD, and the benefits of Mg supplements in such patients.

The review is based on a comprehensive search of relevant articles in databases such as PubMed, Medline, PubMed Central, and Google Scholar. The search terms included magnesium, serum magnesium, the role of magnesium in cardiovascular functions, the role of magnesium in renal functions, serum magnesium and CVDs, serum magnesium, and CKD, hypomagnesemia in CVD, hypomagnesemia in CKD, magnesium supplements, and benefits of magnesium supplements in CVD and CKD. Since we are not doing a systematic review, we have not identified all the literature. Therefore, we acknowledge a publication bias.

## Review

Magnesium

Mg is the second most abundant intracellular electrolyte [10] after potassium and the fourth most abundant cation in the human body, following sodium (Na^+^), potassium (K^+^), and calcium (Ca^2+^). Most of the Mg is intracellular and a vital component of bone minerals. Less than 1% of the total body Mg is extracellular with 30% protein-bound and the rest as free ionized Mg (up to 70%) or mineral complex (less than 10%) [11,12]. Bone contains up to 60%, muscle contains up to 30%, and other soft tissues contain up to 20% of total body Mg. Mg exchange occurs between the compartments such as bone, muscle, and other soft tissues. The primary regulation of serum Mg concentration occurs renally with about 95% tubular reabsorption [12-14]. The major sources of dietary Mg include green vegetables, whole grains, nuts, and seeds. In addition, fruits, fish, and meat contribute to Mg intake, albeit to a lesser extent [15].

Role of Mg in cardiovascular and renal functions

Although Mg is less potent than the organic Ca^2+^ channel blockers, Mg exerts unique and potentially beneficial Ca^2+^ channel antagonistic effects in vascular smooth muscle cells (VSMCs) by acting on voltage-, receptor-, and leak-operated membrane channels. Mg can block Ca^2+^ influx and efflux at vascular membranes; reduce resistance in peripheral and cerebral blood vessels; alleviate cerebral, coronary, and peripheral vasospasm; and reduce arterial blood pressure [16].

Several experimental studies have widely hypothesized the mechanisms of inhibition of vascular calcifications. Brake et al. (2017) outlined two leading hypotheses regarding Mg and vascular calcification. First, the passive interference hypothesis is that Mg may bind to phosphate and slow down the growth of calcium phosphate crystals in the bloodstream leading to indirect interference with the deposition of Ca^2+^ phosphate in the vessel walls. Second, the active cellular modulation hypothesis is that the process of VSMC transdifferentiation is triggered by reduced levels of circulating inhibitors of vascular calcification, along with elevated inorganic phosphate (Pi) levels and the formation of amorphous Ca²⁺-Pi particles (ACP) in the bloodstream. This VSMC transdifferentiation is further expedited by the osteogenic genes’ expression and amplified by the release of exosomes and apoptotic bodies by VSMCs. Mg potentially prevents this process via different pathways both on the level of initiation and acceleration of VSMC calcification. Mg inhibits osteogenic conversion by preventing the loss of calcification inhibitors such as BMP-7, matrix Gla protein, and osteopontin [17]. In addition, Mg reverses the osteogenic transformation of VSMCs by inhibiting the Wnt/β-catenin signaling pathway [18].

Mg also plays a role in regulating blood pressure and endothelial function. Elevated serum Mg levels are linked to enhanced endothelial function in individuals with CKD, as evidenced by heightened flow-mediated dilation of the brachial artery [19,20]. Conversely, in individuals without CKD, higher serum Mg levels are correlated with a reduced risk of developing hypertension [21]. Mg interacts with the calcium-sensing receptor (CaSR), which is not only found in the parathyroid gland but also expressed in VSMCs. The activation of CaSR by calcimimetics has been shown to suppress the calcification of VSMCs in laboratory studies [22,23]. Mg might produce analogous effects.

Studies have demonstrated that Mg can increase the expression of CaSR in the parathyroid gland, leading to a reduction in parathyroid hormone (PTH) secretion. In addition, in patients undergoing hemodialysis, higher serum Mg levels were linked to lower serum PTH concentrations. However, the impact of Mg on the CaSR of VSMCs has yet to be investigated [24,25]. Within VSMCs, Mg hinders the entry of calcium through L-type Ca^2+^ channels, thereby diminishing vascular tone [16]. On the endothelial front, Mg plays a critical role in acetylcholine-induced vasodilation, facilitated by the release of vasorelaxant factors like nitric oxide (NO). This vasodilatory response is contingent upon adequate Mg levels and is further augmented by elevated Mg concentrations in laboratory settings [26,27].

Renal fibrosis (RF) is a hallmark feature of CKD, characterized by the activation of renal interstitial fibroblasts and excessive accumulation of extracellular matrix (ECM). Mg protects the kidneys, retards renal failure progression, and delays CKD by modulating inflammation, oxidative stress, and fibrosis-related pathways [28]. Mg modulates TRPM6/7 channels, leading to increased intracellular Mg levels. This inhibits Nrf2 activation via the PI3K/Akt and PKC pathways, thereby reducing oxidative stress and inflammation.

Mg promotes the secretion of the klotho protein, which has potent anti-fibrotic effects and contributes to improving RF by counteracting ECM deposition. Throughout CKD progression, inflammation and oxidative stress play crucial roles. Mg inhibits the differentiation of inflammatory cells, downregulates the production of inflammatory cytokines, and reduces reactive oxygen species (ROS) production and oxidative stress. Mg also suppresses the TLR-4/NF-κB signaling pathway, further mitigating inflammation and oxidative stress. Some antifibrotic effects of Mg may relate to its antagonism of intracellular Ca^2+^. Mg also regulates apoptosis, safeguarding renal tubular function [28-31].

Hypomagnesemia in CVDs

As Mg is primarily located on the inner surface of cell membranes, it contributes to the regulation of permeability of the cell membranes to Na^+^ and K^+^ [32]. Mg can activate the Na^+^-K^+^-ATPase pump, which regulates the movement of Na^+^ and K^+^ ions across cell membranes. Fluctuations in Mg levels can affect membrane permeability, causing K^+^ depletion and increasing intracellular concentrations of Ca^2+^ and Na^+^. The increased intracellular Ca^2+^ can lead to hypertension, vasospasm, and increased sensitivity to vasoconstrictors [33]. Shibutani et al.'s study on 380 Japanese junior high school students revealed a correlation between elevated systolic blood pressure, family history of hypertension, and lower Mg levels in serum and erythrocytes. This indicates a potential role of Mg deficiency in high blood pressure, especially among those with a family history of hypertension, suggesting a link between genetic predisposition to hypertension and Mg deficiency [34].

Human studies emphasize the connection between Mg and cardiomyopathy. Patients with hypoparathyroidism can develop cardiomyopathy, which responds positively to Mg supplementation [35]. Szabo et al. found that a slight decrease in extracellular Mg levels from 1.2 to 0.8 mM did not affect vasomotor tone when the endothelium was intact. Conversely, when the endothelium was disrupted, this modest reduction in Mg led to an elevation in vascular tone. These findings indicate that Mg affects human vasomotor tone through endothelium-derived relaxing factors rather than directly altering smooth muscle tone. In addition, Mg deficiency seems to contribute to endothelial dysfunction and, consequently, atherosclerosis [36]. Amighi et al. studied 323 individuals with symptomatic peripheral artery disease and intermittent claudication to assess the prognostic relevance of serum Mg levels and the risk of neurological events. They found that patients with lower serum Mg levels (<0.76 mmol/L) had a significantly higher risk of neurological events compared to those with higher levels (>0.84 mmol/L). This suggests a correlation between low serum Mg levels and a heightened risk of neurological events, such as ischemic stroke and carotid revascularization [37]. In another study involving 40 patients with acute ischemic strokes, a link was observed between low serum Mg concentration and the severity of neurological deficit at 48 hours post-stroke onset. Patients with lower Mg levels experienced more severe paresis [38].

Mg deficiency might lead to a continual release of Ca^2+^ from the sarcoplasmic reticulum through Ryanodine receptors in the heart's relaxation phase. This phenomenon, termed the "leaky ryanodine receptor" hypothesis, is associated with impaired diastolic relaxation and diastolic heart failure [39]. As Mg serves as a co-factor in enzymatic reactions linked to ATP, its deficiency can disrupt the process of Ca^2+^ reuptake into the sarcoplasmic reticulum through the ATP-driven SERCA pump. This disruption may lead to inadequate relaxation during diastole, contributing to diastolic heart failure. Furthermore, the delayed decay of cytoplasmic Ca^2+^ could diminish Ca^2+^ release in the subsequent cardiac cycle, resulting in decreased contractility and systolic heart failure. Moreover, optimal Mg levels are necessary for ATP-dependent muscle fiber contraction. Conversely, excessive Mg can have adverse effects by reducing Ca^2+^ influx through L-type Ca^2+^ channels, which play a crucial role in excitation-contraction coupling [40]. Mg plays a direct role in maintaining endothelial function and low Mg levels contribute to endothelial dysfunction by creating a proinflammatory, prothrombotic, and proatherogenic environment. Mg offers protection against atherosclerosis and promotes the growth of collateral vessels in chronic ischemia [41].

Hypomagnesemia in CKD

In a cross-sectional study involving 5,126 patients at various stages of CKD, hypomagnesemia emerged as the prevalent electrolyte anomaly. Proteinuria in CKD prompts renal Mg loss, thereby culminating in hypomagnesemia [42,43]. In individuals with CKD dietary K^+^ restriction can lead to hypomagnesemia as K^+^-rich foods are typically abundant in Mg. Factors such as diabetes mellitus and the use of loop and thiazide diuretics can increase urinary Mg loss. Furthermore, impaired urinary Mg reabsorption stemming from tubular dysfunction or interstitial fibrosis plays a role [44]. Low Mg levels are linked to an increased risk of developing CKD or progressing to ESRD in individuals with an estimated glomerular filtration rate (eGFR) greater than 60 mL/min/1.73 m² [45].

In a retrospective cohort study involving individuals with diabetic kidney disease, researchers found that hypomagnesemia was associated with a 2.12-fold higher risk of progression to ESRD. This highlights the importance of monitoring Mg levels in patients with diabetic kidney disease to potentially mitigate the risk of kidney disease progression [9]. In a cohort study of patients with non-diabetic CKD, researchers found that high serum phosphate levels were associated with an increased risk of progression to ESRD only when their serum Mg levels were low. Interestingly, the risk was mitigated when their serum Mg levels were high. This highlights the importance of considering both Mg and phosphate levels in managing CKD patients to optimize kidney health [46].

Benefits of Mg supplements in CVDs and CKD

Mg exhibits vasodilatory, anti-inflammatory, anti-ischemic, and antiarrhythmic properties. Consequently, it is considered a potentially valuable therapeutic agent in cardiovascular medicine. Numerous studies have highlighted the involvement of Mg in the pathogenesis of CVD within the general population [47,48]. Low serum Mg level increases the risk of CVD [49,50], and Mg supplement is associated with a reduced incidence of CVD [51,52].

Mg infusion offers relief for individuals with vasospastic angina by promoting general coronary artery dilation and alleviating acetylcholine-induced coronary spasms. It also reduces chest pain severity and ST-segment deviations during spasms. Moreover, after Mg infusion, there is a notable improvement in the percentage change of spastic segment diameter during coronary spasms [53]. Several studies in humans have found an inverse relationship between serum or dietary Mg levels and the occurrence of vascular calcification. This includes conditions like coronary artery calcification (CAC), aortic calcification (AC), and vascular measurements, such as intima-media thickness (IMT) and pulse wave velocity (PWV), which can partially reflect vascular calcification [54-56].

Supplementing with dietary Mg has been found to lower plasma glucose levels in individuals with pre-diabetes and hypomagnesemia [57]. In addition, it enhances insulin sensitivity, lowers fasting glucose levels, and reduces HbA1c concentrations in patients diagnosed with type 2 diabetes mellitus (T2DM) [58]. Hypomagnesemia was significantly linked to and independently predicted the progression to ESRD in patients with T2DM and diabetic nephropathy, but this association was not observed in those with non-diabetic CKD [9]. In patients with CKD, common comorbid conditions include vascular calcification, hypertension, diabetes, and diabetic nephropathy. These factors are all potentially influenced by Mg levels, and there is growing evidence supporting the beneficial effects of Mg supplementation and maintaining slightly elevated Mg levels [59].

In CKD patients, Mg can be administered via oral supplements, Mg-enriched diet, and adjusting dialysate Mg concentration for those undergoing hemodialysis or peritoneal dialysis. Among the available oral Mg formulations, such as Mg citrate, Mg oxide, Mg hydroxide, Mg sulfate, and Mg carbonate, Mg citrate stands out due to its superior bioavailability. In addition, Mg carbonate is commonly prescribed to manage hyperphosphatemia because of its ability to bind phosphate [60]. A randomized clinical trial demonstrated that oral Mg oxide effectively slowed the progression of coronary artery calcification (CAC) in patients with stage 3-4 CKD. Remarkably, this effect was observed irrespective of the severity of baseline CAC. The study findings suggest that Mg supplementation could be a valuable strategy for preventing CAC progression in CKD patients [61]. Several case reports have described that long-term intramuscular and subcutaneous Mg administration can effectively restore Mg deficiency but cause local pain and irritation [62-64].

## Conclusions

Mg is important to maintain and protect the cardiovascular and renal functions. Higher Mg concentrations and Mg supplementation have been found to be protective against CVDs and CKD. Mg safeguards the kidneys, slows down the progression of renal failure, and delays CKD progression by regulating pathways associated with inflammation, oxidative stress, and fibrosis. Mg has the potential to impact the frequent comorbid factors, such as vascular calcification, hypertension, diabetes, and diabetic nephropathy linked to higher mortality rates in patients with CKD. Studies suggest that timely Mg supplementation may benefit patients with CVDs and CKD. However, further prospective randomized controlled trials are needed to fully understand the therapeutic impact of Mg on CVDs and CKD, to establish the impact of Mg supplementation on relevant clinical outcomes, and to define personalized target ranges for serum Mg concentrations in patients with CVD and CKD.
